# Deciphering interaction mechanisms in heat treatment time-modulated whey protein isolate-*Lycium barbarum* polysaccharide complexes: perspectives from structural-functional evolution

**DOI:** 10.1016/j.fochx.2025.103440

**Published:** 2025-12-22

**Authors:** Shaomin Zheng, Fangyan Huang, Bingqiang Wang, Jinjing Yang, Huan Han, Leiwen Xiang, Hailin Wang

**Affiliations:** a College of Food and Bioengineering, Fujian Polytechnic Normal University, Fuqing, Fujian, China; b Fujian Province-Indonesia Marine Food Joint Research and Development Center, College of Food and Bioengineering, Fujian Polytechnic Normal University, Fuqing, Fujian, China; c College of Food Science, Fujian Agriculture and Forestry University, Fuzhou, China; d Zhe Jiang Institute of Tianjin University, Shaoxing, Zhejiang, China

**Keywords:** Heat treatment time, WPI-LBP complex, Function, Structure, Interaction

## Abstract

This study investigated the temporal evolution of the structure-function relationship in whey protein isolate-*Lycium barbarum* polysaccharide (WPI-LBP) complexes during 95 °C heating to identify optimal processing windows and underlying interaction mechanisms. The degree of grafting (DG) peaked at 60 min (37.43 %), confirmed electrophoretically. Solubility, emulsifying activity index (EAI), and thermal stability rose then fell with heating, while surface hydrophobicity (H_0_) and free sulfhydryl (SH) content were inversely correlated, with critical points at 30 and 60 min. Circular dichroism (CD) and X-ray diffractometer (XRD) analysis showed time-dependent changes in secondary and crystal structures. Microscopy revealed and water state analysis showed that moderate heating for 30 min produced complexes with smooth surfaces and uniform aggregates. Moreover, the results suggest that WPI-LBP complexes formation is primarily driven by covalent bonds and hydrophobic interactions, supplemented by hydrogen bonds and electrostatic forces. These findings offer theoretical guidance for utilizing WPI-LBP complexes in food applications.

## Highlights

• Whey protein isolate (WPI)-*Lycium barbarum* polysaccharide (LBP) complexes formed.
• Heat durations govern functional-structural characteristics of WPI-LBP complexes.
• Optimal heating time maximizes solubility and emulsifying activity index.
• Multispectral/microscopy: Heat-duration WPI-LBP structural modification.
• Covalent, H-bond, hydrophobic, electrostatic forces dominate via heat duration.

## 1 Introduction

Proteins and polysaccharides, as two essential components of food, play a significant role in thickening, forming, and texturizing food systems (Wang et al., 2025). Studies have shown that different reaction conditions (such as temperature, time, pH, protein-to-polysaccharide ratio, and salt ions) can affect the interactions between proteins and polysaccharides, altering their functional properties and structures (Babu et al., 2024; Xu et al., 2019). Therefore, understanding the changes in their functional properties and structures under various reaction conditions is crucial for optimizing the application of protein-polysaccharide complexes in fields such as food and pharmaceuticals.

WPI is a byproduct of cheese production, composed of over 90 % whey protein, primarily including β-lactoglobulin, α-lactalbumin, and bovine serum albumin (Wang et al., 2025; Zhu et al., 2025). WPI is widely used in the food industry due to its excellent emulsifying and gelling properties (Zhu et al., 2025). However, as its application range expands, its original functional properties and structure can no longer meet the demands of new food systems. Studies have shown that the addition of various biopolymers can alter the functional properties and structure of WPI (He et al., 2021). Among these, polysaccharide biopolymers, known for their stability and bioactivities such as antioxidant and antitumor properties, are considered the most direct and effective method for improving protein functionality and structure (He et al., 2021; Wang et al., 2025; Yao et al., 2023). For example, Yao et al. (2023) used mesona chinensis polysaccharides (MCP) to prepare WPI-MCP complexes as O/W emulsion stabilizers. The results showed that the combination of MCP and WPI reduced surface hydrophobicity, increased the apparent viscosity and gel structure of the emulsion, and enhanced its oxidative stability. Additionally, studies have shown that the multiple hydroxyl groups in polysaccharide molecules enable them to bind to proteins through hydrogen bonding and electrostatic interactions, and these non-covalent interactions help enhance the functional and nutritional properties of proteins (Alrosan et al., 2024).

*Lycium barbarum*, used both as medicine and food, are primarily cultivated in subtropical regions such as China, Korea, Southeast Asia, and parts of Europe (Ni et al., 2025; Yao, 2025; Zhang & Wang, 2025). They contain numerous bioactive components, including polysaccharides, polyphenols, betaine, and carotenoids, which confer a wide range of regulatory, nutritional, and therapeutic properties (An et al., 2024). Among these, *Lycium barbarum* polysaccharides (LBP), composed of various acidic heteropolysaccharides, are considered the most important and extensively studied bioactive components in *Lycium barbarum* (He et al., 2021; Wang, Rao, et al., 2024). Extensive research has been conducted on the isolation, purification, structure, and bioactivity of LBP (Masci et al., 2018). Crucially for this study, LBP's unique structural properties translate into compelling functional interactions with proteins. Recently, researchers have focused on the rheological behavior of LBP and its regulatory effects on the functional properties and structure of proteins (He et al., 2021; Wang, Lu, et al., 2022; Wang, Rao, et al., 2024; Xing et al., 2024). Studies have shown that adjusting LBP concentration can effectively regulate the gel behavior and structure of WPI (He et al., 2021), gelatin (Wang, Rao, et al., 2024), and soy protein (Xing et al., 2024), with appropriate concentrations of LBP significantly enhancing the gel performance of these proteins. Therefore, we selected LBP to leverage its established potential for creating diverse and functional complexes with proteins. However, current research on LBP-protein interactions mainly focuses on regulating gel performance, with less attention on its effects on other protein functional properties (such as solubility and emulsification) and related structures. Moreover, while extensive research on protein-polysaccharide interactions has focused on reaction temperature, pH, ionic strength, among other factors, systematic investigations into processing duration remain relatively limited (Babu et al., 2024). Nevertheless, previous research has shown that heating time should be carefully optimized and kept as short as possible in order to prevent the reaction from reaching advanced stages, thereby limiting excessive polymerization and browning, as well as impairments in functionality and structure (Qu et al., 2018). For instance, Qu et al. (2018) reported that varying the heat treatment time for rapeseed protein isolate and dextran conjugates directly influenced the degree of grafting, resulting in structural changes such as increased molecular weight and a more porous surface, along with improved functional properties including solubility, emulsifying capacity, and thermal stability. Therefore, understanding the temporal evolution of heat-induced conjugation is crucial, as it not only advances our knowledge of the dynamic structure-function relationships but also provides a theoretical basis for modulating functionalities by controlled reaction progression. Our previous research indicated that LBP concentration significantly alters the functional and structural properties of WPI, with the addition of 1 % LBP proving particularly effective in enhancing solubility, emulsification, and digestibility (Wang et al., 2025). Building upon this foundation, we hypothesize that heat treatment time acts as a critical, non-linear modulator of the dominant interaction forces in WPI-LBP complexes, with an optimal window existing where covalent grafting (Maillard reaction) and non-covalent interactions synergistically enhance functionality before degradation processes dominate. To test this hypothesis, this study aims to delineate the temporal evolution of the structure-function relationship in WPI-LBP complexes during heat treatment at 95 °C, specifically to identify optimal time points for key functional properties and to infer the underlying interaction mechanisms. This will be achieved through a comprehensive strategy that correlates evolving functional properties with multi-faceted structural characterization. These results will provide useful information for the application of WPI-LBP complexes in the food and related industries.

## 2 Materials and methods

### 2.1 Materials and reagents

LBP was purchased from Xi'an Xin Tuo Biological Products Co., Ltd. (China), containing 62.82 % neutral sugars, 22.53 % uronic acid, and 4.58 % protein. WPI was obtained from Hilmar Ingredients (USA) as product Hilmar 9400, with a manufacturer-specified composition of 90.0 % protein (as is), 0.1 % lactose, 0.6 % fat, 4.25 % moisture, and 2.7 % ash. Standard monosaccharides was obtained from Sigma-Aldrich (USA). Both O-phthalaldehyde (OPA) and Ellman's reagent were purchased from Shanghai Macklin Biochemical Co., Ltd. (China). 8,1-Anilinonaphthalenesulfonate (ANS) was sourced from Sigma-Aldrich Co., Ltd. (USA). All other reagents were of analytical grade.

### 2.2 Determining the molecular weight (Mw) and monosaccharide composition of LBP

The *Mw* of LBP was determined using a PL-GPC50 system equipped with a refractive index detector. Separation was performed on two PLgel aquagel-OH Mixed-M columns (7.5 × 300 mm, 8 μm) at 40 °C, with an aqueous mobile phase (0.02 mol/L NaNO_3_ and 0.01 mol/L NaH_2_PO_4_, pH 7.0) at a flow rate of 1.0 mL/min. Dextran standards were used to establish the calibration curve.

Monosaccharide composition was analyzed by ion chromatography (ICS 5000+ system). Samples were first hydrolyzed with 2.0 mol/L trifluoroacetic acid at 121 °C for 2 h, then derivatized and injected into a CarboPac PA20 column (150 × 3.0 mm, 10 μm) maintained at 30 °C. Elution was carried out at 0.5 mL/min using a gradient of water, NaOH (0.1 mol/L), and a NaOH/NaAc mixture.

### 2.3 Preparation of WPI-LBP complexes

Sample preparation was performed as described by Wang et al. (2025) to produce WPI-LBP complexes under different heat treatment durations; the specific complexes used were those exhibiting optimal functional properties. A 2.0 % (*w*/*v*) LBP solution was prepared using distilled water and kept at 4 °C overnight. An appropriate amount of WPI was added to distilled water, stirred at 300 r/min for 2 h, and stored at 4 °C overnight to obtain a 4.0 % (w/v) WPI solution. These two solutions were mixed at a 1:1 (*v*/v) ratio and stirred for 30 min to ensure uniformity. The pH of the sample solution was adjusted to 7 using 0.1 mol/L NaOH or HCl. The solution was then transferred to blue-cap bottles, sealed, and heat-treated in a water bath at 95 °C for 0, 15, 30, 60, 120, and 180 min. A control group was prepared by mixing the 4.0 % WPI solution with distilled water at a 1:1 ratio. After the reaction, the solutions were rapidly cooled to room temperature using ice water and then freeze-dried for storage.

### 2.4 Determination of degree of grafting (DG)

The determination of DG was based on the method of Wang et al. (2025), using the OPA method to measure the DG of WPI-LBP complexes prepared with different heat treatment times. Briefly, 2 mg/mL solutions of various WPI-LBP complexes were prepared using 0.01 mol/L PBS (pH 7.4). A 200 μL sample solution was mixed thoroughly with 4 mL of OPA reagent and reacted in a water bath preheated to 35 °C for 2 min. A blank was prepared by replacing the sample solution with 200 μL of distilled water, reacting at 35 °C for 2 min. The absorbance of each WPI-LBP complex sample was measured at 340 nm and recorded as A_1_. After reacting 200 μL of WPI with 4 mL of OPA, the absorbance was measured at 340 nm and recorded as A_0_. The DG of each complex was calculated using the following formula (1):

(1) $$\mathrm{DG}\;\left(\%\right)=\left(A_{0}-A_{1}\right)/{A_{0}}^{*}100\%$$

### 2.5 Sodium dodecyl sulfate-polyacrylamide gel electrophoresis (SDS-PAGE)

SDS-PAGE was performed according to Wang et al. (2025) using 12 % separating and 5 % stacking gels. Samples were prepared at 2 mg/mL in 0.02 mol/L PBS (pH 7.4), treated with loading buffer, heated to 95 °C for 5 min, cooled to room temperature, and centrifuged at 10,000 rpm for 5 min. Supernatants (20 μL) were loaded onto gels. Electrophoresis initiated at 80 V through stacking gels, with voltage increased to 120 V when bromophenol blue reached the separating gel interface. Proteins were visualized by sequential staining with Coomassie Brilliant Blue R-250 and Schiff's reagent.

### 2.6 Determination of functional characteristics of WPI-LBP complexes

#### 2.6.1 Determination of surface hydrophobicity (H_0_)

The H_0_ of WPI-LBP complexes was determined according to the ANS-binding method described by Wang et al. (2025) and Li, Lai, et al. (2024). Briefly, a series of sample solutions with concentrations ranging from 0.0625 to 1 mg/mL were prepared in 0.01 mol/L PBS (pH 7.4) via gradient dilution. For the assay, a 5 mL aliquot of each concentration was mixed with 25 μL of an 8 mmol/L ANS solution. After vortexing, the mixture was incubated in the dark for 20 min. The fluorescence intensity was then measured using an FL970 fluorescence spectrophotometer (Shanghai Techcomp Scientific Instruments Co., Ltd., China) at excitation and emission wavelength of 379 nm and479 nm, respectively, with a pure PBS solution serving as blank. The H_0_ value was defined as the initial slope of the plot of fluorescence intensity against sample concentration.

#### 2.6.2 Determination of solubility

The solubility of WPI and WPI-LBP complexes was determined following the method of Wang et al. (2025). Briefly, sample solutions (2 mg in 0.01 mol/L PBS) were centrifuged at 10,000 r/min for 20 min. The supernatant of each sample was collected, and the protein content in the supernatant was measured using the Folin-phenol method.

#### 2.6.3 Determination of emulsification properties

The EAI and ESI of WPI and WPI-LBP complexes were determined with the method of Wang et al. (2025). Briefly, emulsions were prepared by homogenizing 9 mL of each sample solution (2 mg/mL in 0.01 mol/L PBS, pH 7.4) with 3 mL of soybean oil, corn oil, or canola oil, respectively, at 15000 r/min for 3 min using an S-35 K handheld homogenizer (Lepard Scientific Instruments (Beijing) Co., Ltd., China). Immediately after homogenization, 50 μL of the emulsion was sampled from a point approximately 5 mm below the surface and promptly diluted with 5 mL of 0.1 % SDS solution. The absorbance of the diluted emulsion was measured at 500 nm immediately (A_0_) and after 30 min (A_30_), using a 0.1 % SDS solution as the blank. The EAI and ESI were then calculated using the following formulas (2) and (3):

(2) $$\mathrm{EAI}\;\left(m^{2}/g\right)=\left(2^{*}2.303^{*}{A_{0}}^{*}\mathrm{DF}\right)/\left(\theta^{*}\varphi^{*}C^{*}1000\right)$$

(3) $$\mathrm{ESI}\;\left(\min\right)=\left({A_{0}}^{*}30\right)/\left(A_{0}-A_{30}\right)$$

where DF is the dilution factor (100); φ is the cuvette path length (cm); θ is the oil phase volume fraction (0.25); and c is the sample concentration (g/mL).

### 2.7 Low-field nuclear magnetic resonance (LF-NMR)

Aliquots (3.0 g) of the liquid WPI-LBP complex solutions (the pre-freeze-dried samples from Section 2.3) were placed in vials and stored at 4 °C for over 12 h prior to LF-NMR analysis. Water distribution states were analyzed using an MicroMR12-040 V LF-NMR analyzer (Niumag Co., China) under the following parameters: spectrometer frequency (sf) = 12 MHz (magnetic field strength = 0.28 T); CPMG_Q pulse sequence; spectral width (SW) = 250 kHz; number of scans (NS) = 4; sampling points (TD) = 2,250,070; echo time (TE) = 0.5 ms; echo number (NECH) = 18,000.

### 2.8 Structural characteristics of WPI-LBP complexes

#### 2.8.1 Free sulfhydryl (SH) content

The determination of free SH content followed the method of Li et al. (2025), with slight modifications. A Tris-glycine buffer with 8 mol/L urea (containing 0.086 mol/L Tris, 0.09 mol/L glycine, and 4 mmol/L EDTA, pH 8.0) was used to prepare 5 mg/mL solutions of WPI and various WPI-LBP complexes. The solutions were then centrifuged at 6000 r/min for 10 min, and the supernatant was collected. A 5 mL portion of the supernatant was mixed with 50 μL of Ellman's reagent (4 mg/mL), thoroughly mixed, and allowed to react in the dark for 15 min. The absorbance at 412 nm was measured using a Multiskan SkyHigh microplate reader (Thermo scientific, USA), with a solution without the sample as the blank. The free SH content was calculated using the following formula (4):

(4) $$\mathrm{SH}\;\text{content}\;\left(\text{μmol}/g\right)=\left(73.53\times A_{412}\right))/c$$

A_412_ is the absorbance value of the sample at 412 nm; c is the concentration of the sample (mg/mL).

#### 2.8.2 Circular dichroism (CD)

A 0.01 mol/L PBS (pH 7.4) solution was used to prepare 0.5 mg/mL solutions of WPI and various WPI-LBP complexes. The CD spectra of each sample were scanned using a J-1700 CD spectropolarimeter (Jacso, Tokyo, Japan) in the range of 190–260 nm, with a scanning speed of 100 nm/min. The secondary structure of each sample was calculated from the obtained CD spectra using Yang's algorithm.

#### 2.8.3 X-ray diffractometer (XRD)

A suitable amount of freeze-dried WPI and various WPI-LBP complexes were placed in the sample holder. The XRD patterns of each sample were measured using an Ultima IV XRD (Rigaku, Japan) in the range of 5° to 60°, with a scanning rate of 2°/min. The current and voltage were set to 40 mA and 40 kV, respectively.

#### 2.8.4 Scanning electron microscopy (SEM)

An appropriate amount of freeze-dried WPI and different WPI-LBP complexes were mounted on double-sided tape and gold-coated. The microstructure of each sample was examined and imaged using a Sigma 360 SEM (Zeiss, Germany), with an acceleration voltage of 3.0 kV.

#### 2.8.5 Atomic force microscopy (AFM)

Samples prepared in Section 2.3 were diluted 100 times using ultrapure water preheated for the same duration at the same temperature. After thorough mixing, the solution was filtered through a 0.45 μm membrane. Then, 10 μL of the filtrate was dropped onto a fresh mica sheet and air-dried. The nanostructure of each sample was observed and photographed using a Dimension Icon AFM (Bruker, Germany) in tapping mode.

#### 2.8.6 Differential scanning calorimeter (DSC)

The T_d_ of WPI and the WPI-LBP complex was analyzed using a Q20 DSC (TA, USA). Briefly, approximately 1.5 mg of the sample was weighed into an aluminum pan, which was then sealed and placed in the sample chamber. The measurement was performed by heating the sample from 30 °C to 150 °C at a constant rate of 5 °C/min under a nitrogen purge flow of 100 cm^3^/min.

### 2.9 Statistical analysis

Statistical analyses were performed with GraphPad Prism and SPSS software. The experiment was independently repeated three times, with results expressed as means ± SD. Significant differences (*p* < 0.05) were assessed by one-way ANOVA coupled with Duncan's multiple range tests.

## 3 Results and discussion

### 3.1 Analysis of the Mw and monosaccharide composition of LBP

As summarized in Table 1, LBP was characterized as a heteropolysaccharide with a molecular weight of 18.92 × 10^3^ Da, composed of arabinose, rhamnose, galactose, glucose, xylose, galacturonic acid, and glucuronic acid in a molar ratio of 2.44: 0.59: 1.47: 0.78: 0.15: 0.84: 0.17.

**Table 1:** *Mw* and monosaccharide composition of LBP.

Sample	LBP
Mw (Da)	18.92 × 10^3^
*Monosaccharide composition (molar ratio)*	
Arabinose	2.44 ± 0.09
Rhamnose	0.59 ± 0.11
Galactose	1.47 ± 0.14
Glucose	0.78 ± 0.01
Xylose	0.15 ± 0.01
Galacturonic acid	0.84 ± 0.09
Glucuronic acid	0.17 ± 0.02

### 3.2 DG analysis

The DG value is one of the main indicators for evaluating the degree of reaction between proteins and polysaccharides in the Maillard reaction (Zhong et al., 2019). The DG values of WPI-LBP complexes with different heat treatment times are listed in Table 2. It can be seen that the DG value of WPI-LBP complexes increases initially and then decreases with longer heat treatment times. The DG value of the untreated WPI-LBP mixture with 1 % LBP was 10.41 %, possibly due to grafting reactions occurring between WPI and LBP at room temperature, even without heat treatment. When the heat treatment times were 15, 30, and 60 min, the DG values of the WPI-LBP complexes significantly increased to 23.49 %, 32.63 %, and 37.43 % (*p* < 0.05), respectively. This result likely because teat unfolds WPI, exposing more amino acid groups that can react with the hydroxyl groups in LBP (Li, Jiang, et al., 2024). Additionally, the rate of increase slowed as a result of the gradual consumption of reactive groups, coupled with steric hindrance from the formed complexes (Li, Lai, et al., 2024). However, when the heat treatment was extended beyond 60 min, the DG value of the WPI-LBP complexes reached a plateau and showed no significant change at 120 min. A pronounced decrease to 29.20 % (*p* < 0.05) was observed when the time was further increased to 180 min. This phenomenon may be related to structural changes or degradation of the WPI-LBP complexes, as similarly reported by Zhang et al. (2015) for oat protein isolate-dextran conjugates. Specifically, prolonged heating may cause either the depolymerization of the covalent bonds formed between WPI and LBP or the degradation of the LBP polysaccharide chains themselves. Alternatively, it could induce excessive protein aggregation, which would sterically hinder or consume available reactive sites. These hypotheses are corroborated by subsequent results from SDS-PAGE (for covalent bond analysis) and CD, SEM, and AFM (for structural aggregation observation).

**Table 2:** DG, H_0_, solubility and free SH content of WPI-LBP complexes with different heat treatment times.

Samples	DG (%)	H_0_	Solubility (%)	SH content (μmol/g)
WPI	–	2646.0 ± 74.1^d^	67.71 ± 2.40^f^	9.66 ± 1.16^d^
0 min	10.41 ± 0.60^a^	2422.3 ± 72.1^c^	51.94 ± 0.67^a^	6.67 ± 0.26^bc^
15 min	23.49 ± 0.77^b^	2241.5 ± 48.3^b^	52.10 ± 0.74^b^	4.97 ± 0.12^a^
30 min	32.63 ± 1.13^d^	1897.9 ± 27.3^a^	58.46 ± 0.75^c^	5.99 ± 0.02^b^
60 min	37.43 ± 0.52^e^	2317.3 ± 41.4^b^	58.98 ± 0.36^c^	6.24 ± 0.04^bc^
120 min	37.13 ± 1.19^e^	2476.6 ± 49.3^c^	57.04 ± 0.47^d^	6.44 ± 0.03^bc^
180 min	29.20 ± 2.24^c^	2431.2 ± 34.0^c^	54.99 ± 0.27^e^	6.83 ± 0.03^c^

Values are mean ± SD. Values with a different letter within the same column are significantly different (*p* < 0.05).

### 3.3 Analysis of SDS-PAGE

**Fig. 1:**
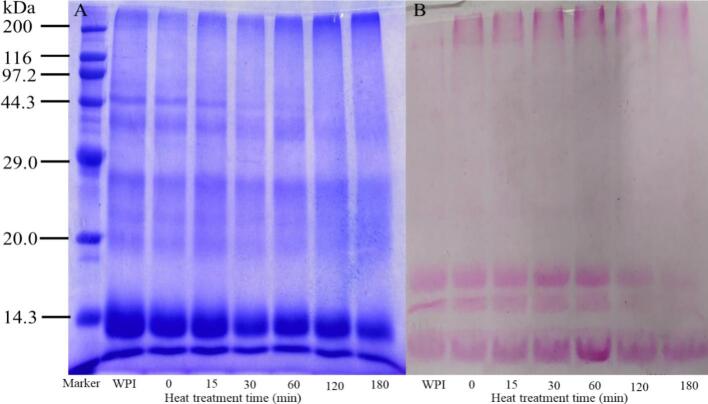
SDS-PAGE analysis of WPI-LBP complexes under varying heat treatment durations. (A) Coomassie Brilliant Blue staining; (B) Schiff's reagent staining. (For interpretation of the references to colour in this figure legend, the reader is referred to the web version of this article.)

SDS-PAGE was employed to validate the impact of varying heat treatment durations on WPI-LBP complex formation, as shown in Fig. 1. In Fig. 1A, it can be observed that untreated WPI without LBP exhibits bands at approximately 14.3, 18.3, 22.5–76.0, and 66.0 kDa, corresponding to α-lactalbumin, β-lactoglobulin, immunoglobulin, and bovine serum albumin, respectively, consistent with previous studies (Sedaghat Doost et al., 2020; Wang et al., 2025). After adding 1 % LBP, these bands faded with increasing heat treatment time, while bands at the interface between the separating and stacking gels became more pronounced. This indicates that with longer heat treatment, WPI and LBP form complexes through the Maillard reaction, and the content of high-molecular-weight complexes increases (Li, Lai, et al., 2024; Wang et al., 2025). As shown in Fig. 1B, the red bands at the interface between the separating and stacking gels became wider and more intense, further supporting the results of Coomassie Brilliant Blue staining. However, it is noteworthy that when the heat treatment time increased to 120–180 min, the red bands of WPI-LBP complexes between 14.3 and 20.0 kDa gradually disappeared. These observations provide further support for the DG results, suggesting that prolonged heat treatment may cause depolymerization of the covalent bonds between WPI and LBP or degradation of the LBP polysaccharide chains.

### 3.4 Analysis of functional characteristics of WPI-LBP complexes

#### 3.4.1 H_0_ analysis

H_0_ represents the number of hydrophobic groups exposed on the surface of protein molecules and can be used to evaluate protein conformational changes (Zhong et al., 2019). The effects of different heating times on the H_0_ of WPI-LBP complexes are listed in Table 2. It can be seen that, compared to WPI without LBP, the H_0_ of the WPI-LBP mixture containing 1 % LBP and not heat-treated significantly decreased from 2646.0 to 2422.3 (*p* < 0.05). The observed decrease in H_0_ for the unheated mixture is most likely attributed to non-covalent interactions between LBP and WPI, such as electrostatic complexes, hydrogen bonding, or hydrophobic interactions, which could indeed induce structural changes in WPI and lead to the burial of hydrophobic residues (Li et al., 2025). When the mixture was heated for 15 and 30 min, the H_0_ of WPI-LBP complexes further decreased to 2241.5 and 1897.9, respectively. The decrease in H_0_ heat-induced WPI aggregation from enhanced hydrophobic interactions, which reduce the hydrophobic sites available for ANS binding (Xing et al., 2024). Notably, when the heating time was extended to 60, 120, and 180 min, H_0_ increased to 2317.3, 2476.6, and 2431.2, respectively. This result may be due to increased exposure of WPI hydrophobic regions with prolonged heating, leading to an increase in H_0_. However, the H_0_ of WPI-LBP complexes heated for 180 min did not differ significantly from that of samples heated for 120 min. This may be because continued heating promotes the reformation of aggregates from degraded WPI polypeptide chains, causing some exposed hydrophobic groups to be re-embedded inside the molecules (Laligant et al., 1991), resulting in no significant change in H_0_.

#### 3.4.2 Solubility analysis

Solubility is one of the most fundamental functional properties of proteins and has a crucial impact on other functional characteristics (Li, Jiang, et al., 2024). The effects of different heating times on the solubility of WPI-LBP complexes are listed in Table 2. It can be seen that the addition of LBP and heat treatment reduce the solubility of WPI to varying degrees. Compared to WPI without LBP, the solubility of the WPI-LBP mixture containing 1 % LBP and not heat-treated significantly decreased from 67.71 % to 51.94 % (*p* < 0.05). This may be due to the addition of LBP interacts with WPI, shielding some of the hydrophobic groups on the WPI surface, thereby reducing its hydrophobic interactions with water molecules and decreasing its solubility (Wang et al., 2025). Compared to the WPI-LBP mixture, the solubility of WPI-LBP complexes initially increases with longer heat treatment times and then decreases. Compared to the untreated WPI-LBP mixture, the solubility of WPI-LBP heated for 15 and 30 min significantly increased to 52.10 % and 58.46 % (*p* < 0.05), respectively. This may be due to the heat-induced grafting of hydrophilic LBP onto WPI, which introduces hydroxyl groups that enhance hydrophilicity and hydrogen bonding, thereby improving water affinity, inhibiting protein aggregation, and ultimately increasing solubility (Li, Jiang, et al., 2024; Qu et al., 2018). However, when the heat treatment time was increased from 30 to 60 min, the solubility of WPI-LBP complexes showed no significant change. When the heating time was extended to 120 and 180 min, the solubility decreased to 57.04 % and 54.99 %, respectively. This decline might result from the excessive unfolding of WPI induced by prolonged heating, which concurrently exposes buried hydrophobic groups (promoting aggregation) and internalizes hydrophilic residues, both of which collectively impair solubility (Qu et al., 2018; Wang et al., 2025). Qu et al. (2018) also reported similar conclusions, noting that the reduction in protein solubility is related to the embedding of hydrophilic amino acid residues into internal sites. The solubility results further support the H_0_ findings.

#### 3.4.3 Emulsification properties analysis

**Fig. 2:**
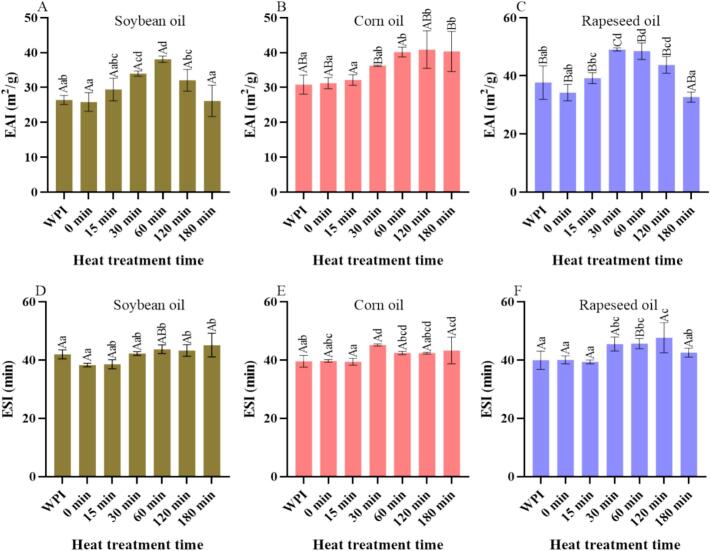
EAI (A–C) and ESI (D–F) of WPI-LBP complexes acorss different heat treatment times and oil types. Note: Different lowercase letters denote significant differences (*p* < 0.05) among samples with different heat treatment times for the same oil type, while different uppercase letters denote significant differences (*p* < 0.05) among different oil types at the same heat treatment time.

EAI describes the ability of proteins to form emulsions, while emulsion stability index (ESI) measures the stability of emulsions over a set time (Dong et al., 2019). The effects of different heating times on the emulsifying properties of WPI-LBP complexes were evaluated by measuring EAI and ESI, as shown in Fig. 2. As shown in Fig. 2A-C, the EAI of emulsions prepared with soybean, corn, and rapeseed oil ranged from 25 to 39, 30–41, and 32–50 m^2^/g, respectively. For unmodified WPI, rapeseed oil yielded a significantly higher EAI than soybean and corn oils (*p* < 0.05). For the WPI-LBP complexes, the EAI of emulsions prepared with corn and soybean oil showed no significant difference at most time points, except at 30 and 180 min where corn oil was higher (*p* < 0.05). Conversely, rapeseed oil produced a significantly higher EAI than both other oils from 0 to 60 min (*p* < 0.05). These differences diminished at longer treatment times, with no significant variations observed among the three oils by 180 min.The EAI of WPI-LBP complex emulsions prepared with soybean and rapeseed oil initially increased with heating time and then decreased. For soybean oil, the EAI peaked at the heating time of 60 min, but there was no significant change compared to the 30 min sample. For rapeseed oil, the EAI peaked at the heating time of 30 min and remained stable thereafter, with no significant differences observed at 60 or 120 min. As shown in Fig. 2D-F, the ESI ranged from 38 to 46 min, 39–46 min, and 39–48 min for emulsions prepared with soybean, corn, and rapeseed oil, respectively. With the exception of the 60-min rapeseed oil sample, which showed a significantly higher ESI (*p* < 0.05), no significant differences were observed among the three oils across other time points. The ESI of the WPI-LBP complex exhibited distinct trends depending on the oil type. For soybean oil, the ESI initially decreased and then increased, peaking at 180 min. In contrast, for emulsions from corn and canola oils, the ESI remained stable initially (0–15 min), followed by an increase and subsequent decrease, with maxima at 30 min and 120 min, respectively.

Previous research indicates that the EAI and ESI of proteins are mainly related to their H_0_ and solubility (Tang et al., 2011). Lower H_0_ indicates weaker hydrophobic interactions between the protein interface and oil droplets, making it harder for droplets to adhere to the surface, which is unfavorable for increasing EAI and ESI; higher H_0_ has the opposite effect (Nooshkam & Varidi, 2020; Tang et al., 2011; Wang et al., 2025). Similarly, lower protein solubility hinders distribution in the aqueous phase, obstructing migration at the oil-water interface, which is unfavorable for increasing EAI and ESI; higher solubility has the opposite effect (Nooshkam & Varidi, 2020; Tang et al., 2011; Wang et al., 2025). Previous results showed H_0_ decreased then increased with heating time, reaching the lowest at 30 min. Solubility results indicated that adding LBP and heat treatment reduced solubility compared to WPI, but solubility increased then decreased with heating time, peaking at 30 and 60 min. Thus, the observed EAI and ESI results may be due to the combined effects of different H_0_ and solubility of WPI-LBP complexes. Furthermore, the distinct fatty acid profiles of the vegetable oils likely contributed to their differential interactions with the WPI-LBP complex. Previous studies have shown that while soybean and rapeseed oils share a high linolenic acid content, they exhibit inverse oleic-to-linoleic acid ratios, with the former containing trace myristic acid and the latter characterized by erucic acid and lower saturation, whereas corn oil contains less linolenic but more oleic acid than soybean oil (Peng et al., 2015). These differences in chain length and unsaturation directly influence the hydrophobic protein-lipid interactions at the oil-water interface, modulating interfacial film rheology and stability, thereby affecting the EAI.

### 3.5 Analysis of the water distribution of WPI-LBP complexes

**Fig. 3:**
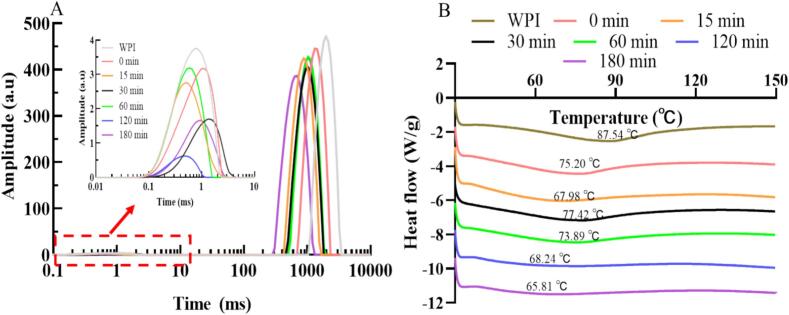
T_2_ mapping (A) and DSC results (B) of WPI-LBP complexes at different heat treatment times.

As a non-destructive and rapid analytical technique, LF-NMR enables effective quantification of water content and distribution states in food proteins, both of which significantly influence their functional properties (Li et al., 2018). The relaxation time (T_2_) spectrum typically reveals three water states: bound water (T_21_, 0–10 ms), immobilized water (T_22_, 10–200 ms), and free water (T_23_, 200–10,000 ms) Li et al., 2018). Fig. 3A illustrates the impact of different heat treatment durations on the T_2_ profiles of WPI-LBP complexes, with corresponding T_2_ and peak areas (S_2_) detailed in Table S1. All samples exhibited distinct relaxation peaks within 0–5 ms and 200–3500 ms. Untreated WPI without LBP showed T_21_ and T_23_ at 0.766 and 1979.167 ms, with peak areas of 1.917 % and 98.083 %, respectively. Incorporation of 1 % LBP increased T_21_ to 1.06 ms while decreasing T_23_ to 1320.088 ms, accompanied by reduced S_21_ (1.527 %) and slightly elevated S_23_ (98.473 %). This shift likely stems from hydrophilic hydroxyl groups in LBP competing with WPI for water molecules, thereby disrupting water-water interactions within the protein network (Wang, Ke, et al., 2022). Compared to untreated WPI-LBP mixtures, complexes subjected to 15, 60, 120, and 180 min heat treatments displayed decreased T_21_ and T_23_ with varying magnitudes, corresponding to diminished S_21_ but increased S_23_. Notably, the 30-min treated complex exhibited paradoxical behavior: T_21_ rose to 1.351 ms (S_21_ = 0.774 %) while T_23_ declined to 1035.322 ms (S_23_ = 99.226 %). These phenomena may originate from heat-induced enhanced interactions between WPI and LBP (e.g., covalent crosslinking, strengthened hydrogen bonding, or hydrophobic effects), which alter the protein network structure and restrict molecular/water mobility, thereby shortening T_2_ relaxation times for both bound and free water. Concurrently, such structural changes might promote the release or redistribution of water molecules from tightly-bound states into free water, consequently reducing S_21_ while increasing S_23_. The observed T_21_ increase in 30-min treated samples is hypothesized to result from a transient competition between marked hydrophilic enhancement from glycosylation (high DG) and extensive protein unfolding (minimal H_0_), which collectively create a unique network that temporarily mobilizes bound water while restricting free water. Notably, the concomitant reduction in T_23_ and increase in S_23_ still indicate decreased overall water mobility with dominant free water fractionation. Heat treatment under these conditions might facilitate the formation of densely ordered network structures, which are proposed to enhance hydrophilicity, thereby potentially improving solubility and emulsification. Solubility, emulsification, SEM and AFM analyses provide corroborative evidence for this interpretation.

### 3.6 Analysis of the structure of WPI-LBP complexes

#### 3.6.1 Free SH content analysis

The active SH groups on the surface of natural protein networks are among the most reactive functional groups in proteins and significantly affect the formation of WPI gels (Jiang et al., 2021; Liu et al., 2017). The effects of different heating times on the free SH content of WPI-LBP complexes are shown in the Table 2. It can be seen that the addition of LBP and different heating times influence the formation of disulfide bonds from free SH, altering the cross-linking between proteins (Li et al., 2019). Compared to WPI without LBP, the free SH content of the WPI-LBP mixture containing 1 % LBP without heat treatment significantly decreased from 9.66 μmol/g to 6.67 μmol/g (*p* < 0.05). This result may be due to LBP reducing the oxidation of WPI, and the steric hindrance induced by LBP limiting the exposure of free SH (Wang, Wang, et al., 2024), altering the protein structure and thus reducing free SH content. When the mixture was heated for 15 min, the free SH content of the WPI-LBP complex further significantly decreased to 4.97 μmol/g (*p* < 0.05), which may be attribute to heat-induced exposed of previously masked sulfhydryl groups and their subsequent participation in disulfide bond formation (Zhao et al., 2020). Notably, compared to a 15-min heat treatment, when the heating time was extended to 30, 60, 120, and 180 min, the free SH content of the WPI-LBP complex increased with heating time, rising to 5.99, 6.24, 6.44, and 6.83 μmol/g, respectively. As heating time increased, LBP promoted protein aggregation through hydrophobic interactions, spatially limiting the formation of disulfide bonds from free SH, thus inhibiting further cross-linking through disulfide bonds (Xing et al., 2024). Additionally, prolonged heating may increase the viscosity of LBP, allowing it to form a gel and interpenetrating structure with proteins, restricting protein chain segment movement, disrupting the conversion between sulfhydryl and disulfide bonds, thereby increasing free SH content (Li et al., 2019). These results further support the H_0_, SEM, and AFM findings.

#### 3.6.2 CD analysis

**Fig. 4:**
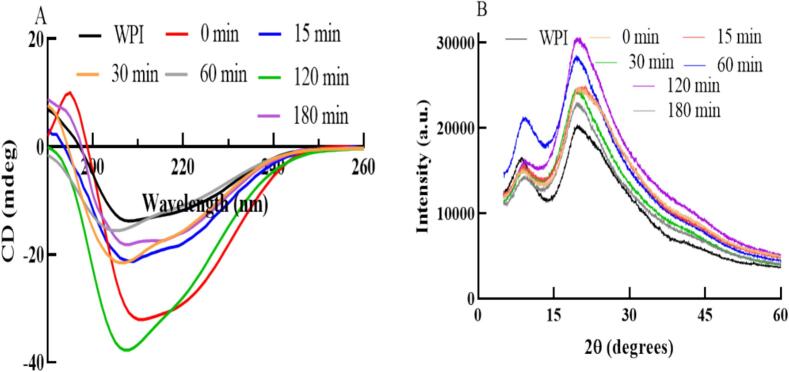
CD (A) and XRD (B) spectra of WPI-LBP complexes with different heat treatment times.

The effects of different heat treatment times on the CD spectra of WPI-LBP complexes are shown in Fig. 4A, with their corresponding secondary structure content listed in Table 3. Fig. 4A indicates that all samples exhibit a characteristic negative peak in the 200–210 nm range, representing the α-helix structure of proteins; the slightly curved peak in the 210–225 nm range corresponds to other secondary structures of proteins (Wang et al., 2025). Additionally, the untreated WPI-LBP mixture shows a distinct positive peak at around 196 nm. These results suggest that different heat treatment times have varying impacts on the CD spectra of WPI-LBP. According to Table 3, compared to WPI without LBP and without heat treatment, the α-helix and β-turn content in the WPI-LBP mixture containing 1 % LBP increased from 18.00 % and 15.00 % to 20.93 % and 20.83 %, respectively, while the β-sheet and random coil content decreased from 29.90 % and 37.15 % to 24.23 % and 34.00 %, respectively. This indicates that the addition of LBP can interact with WPI through covalent or non-covalent bonds, altering its structure. This result further supports the findings of DG, H_0_, solubility, and free SH content. However, when the WPI-LBP mixture containing 1 % LBP was subjected to different heat treatment times, the α-helix content initially decreased and then increased with longer heat treatment. The β-turn and β-sheet content increased or decreased to varying degrees with longer heat treatment, while the random coil content initially increased and then decreased. The critical points for changes in secondary structure were at 30 and 60 min of heat treatment. At 30 min, the α-helix, β-sheet, β-turn, and random coil content of the WPI-LBP complex were 9.25 %, 46.80 % (highest), 5.90 % (lowest), and 38.05 %, respectively, consistent with previous research (Wang et al., 2025), while at 60 min, the content was 8.97 % (lowest), 39.93 %, 11.73 %, and 39.37 % (highest), respectively. Research indicates that the secondary structure of proteins is primarily maintained by intramolecular hydrogen bonds, which are mainly present in the α-helix, β-sheet, and β-turn structures, with fewer in random coils (Jiang et al., 2021; Li, Lai, et al., 2024). From these results, it is evident that different heat treatment times mainly affect the α-helix, β-sheet, and β-turn structures of the WPI-LBP complex, with less impact on random coils. These spectral changes are consistent with significant restructuring of the protein, likely stabilized by a combination of new intermolecular hydrogen bonds and other forces (Li, Lai, et al., 2024). Additionally, electrostatic interactions between LBP and WPI, as noted in previous research (Li, Lai, et al., 2024), can alter the hydrogen-bonding network within WPI molecules, thereby influencing their secondary structure composition.

**Table 3:** Secondary structure content of WPI-LBP complexes with different heat treatment times.

Samples	α-helix (%)	β-sheet (%)	β-turn (%)	Random coil (%)
WPI	18.00 ± 0.57^d^	29.90 ± 2.40^b^	15.00 ± 2.26^d^	37.15 ± 0.35^cd^
0 min	20.93 ± 0.31^e^	24.23 ± 1.31^a^	20.83 ± 0.50^e^	34.00 ± 0.50^a^
15 min	14.03 ± 0.68^c^	33.77 ± 2.82^b^	14.50 ± 1.04^d^	37.70 ± 1.37^d^
30 min	9.25 ± 0.64^ab^	46.80 ± 1.56^d^	5.90 ± 0.99^a^	38.05 ± 1.20^de^
60 min	8.97 ± 1.22^a^	39.93 ± 3.29^c^	11.73 ± 1.40^c^	39.37 ± 0.57^e^
120 min	10.20 ± 0.40^b^	43.30 ± 1.65^cd^	10.80 ± 0.90^bc^	35.77 ± 0.35^bc^
180 min	14.50 ± 0.10^c^	40.77 ± 0.35^c^	9.70 ± 0.20^b^	35.06 ± 0.06^ab^

Values are mean ± SD. Values with a different letter within the same column are significantly different (*p* < 0.05).

#### 3.6.3 XRD analysis

The influence of varying heat treatment durations on the XRD patterns of WPI-LBP complexes is illustrated in Fig. 4B. It shows that all samples have a small diffraction peak around 9° and a stronger one near 20°, indicating their crystalline polymer nature. Compared to WPI without LBP and heat treatment (black line), the WPI-LBP mixture exhibited a noticeable reduction in the diffraction peak intensity at around 9°, while its peak position remained almost unchanged. In the WPI-LBP composites formed after heat treatment, this peak shifted towards higher angles. Its intensity remained at a level similar to that of the mixture for the 15, 30, and 180-min samples, showed little change compared to the untreated WPI for the 60-min sample, and dropped to its lowest value alongside significant peak broadening for the 120-min sample. This suggests that the addition of LBP and varying heat treatment times impact the crystalline structure of WPI-LBP complexes (Wang et al., 2025; Zhang et al., 2022). Previous research indicates that hydrogen bonding, hydrophobic interactions, and electrostatic forces during protein-polysaccharide interactions can influence the crystalline structure of the resulting complexes (Zhang et al., 2022). Thus, the crystalline structure differences in WPI-LBP complexes with different heat treatment times may be related to these interaction forces, aligning with solubility and CD results.

#### 3.6.4 SEM analysis

**Fig. 5:**
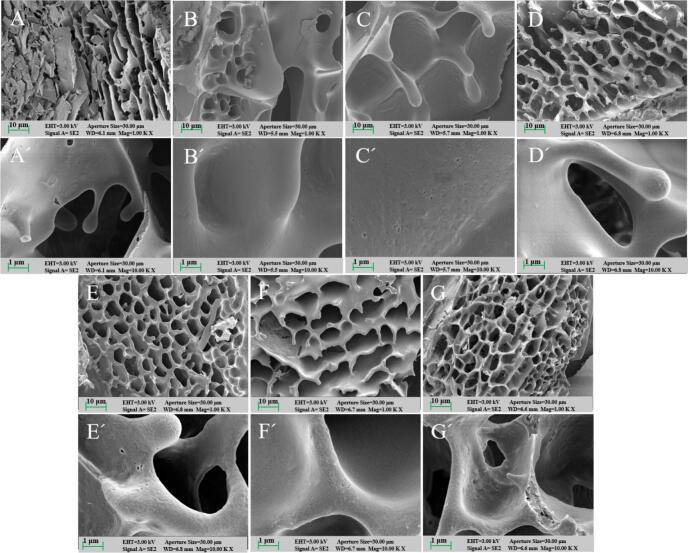
The SEM microstructure of WPI-LBP complexes with different heat treatment times.

The impact of different heat treatment durations on the microstructure of WPI-LBP complexes containing 1 % LBP is shown in Fig. 5. As seen in Fig. 5A and A', WPI without LBP and heat treatment appears smoother and exhibits a dense, compact surface structure. The structure evolved from the initial incorporation of LBP, through the formation of an ordered network, to eventual degradation with prolonged heating. Specifically, the addition of LBP created a rougher, more porous structure with a partially visible network (Fig. 5B and B‘), likely due to hydroxyl groups from LBP competing for water and enhancing hydrogen bonding, thereby stabilizing the gel network (Huang et al., 2024; Wang et al., 2023). Subsequent short-to-moderate heating (15–30 min) facilitated the formation of progressively smoother surfaces and denser, cross-linked networks (Fig. 5C-D and C‘- D'). This structural refinement is consistent with heat-induced unfolding of the WPI structure, which enhances WPI-LBP interactions, leading to an increased DG and the formation of a dense network structure(Liu et al., 2017; Qu et al., 2018). Conversely, extended heating (60–180 min) induced a clear trend of microstructural coarsening. The surface became rougher with depressions (60 min), developed cracks and increased porosity (120 min), and ultimately exhibited significant structural disruption with large depressions and broken cross-links at 180 min (Fig. 5E-G‘). These results indicate that excessively long heat treatment times roughened the WPI-LBP complex structure, making it porous and even causing partial cross-linking damage at 180 min. This structural deterioration is attributed to continuous heating, which over-expands the WPI structure, exposing hydrophobic regions, and consequently leads to protein aggregation and surface depressions (Wang et al., 2025). Additionally, prolonged heat treatment may cause LBP to absorb excess water, forming a continuous hydrogel through hydrogen bonding, and creating an interpenetrating structure with WPI, hindering hydrophobic aggregation and resulting in a loose and disrupted gel network structure (Cao et al., 2022; Huang et al., 2024). This finding further supports the results of H_0_, solubility, SH content, and CD.

#### 3.6.5 AFM analysis

**Fig. 6:**
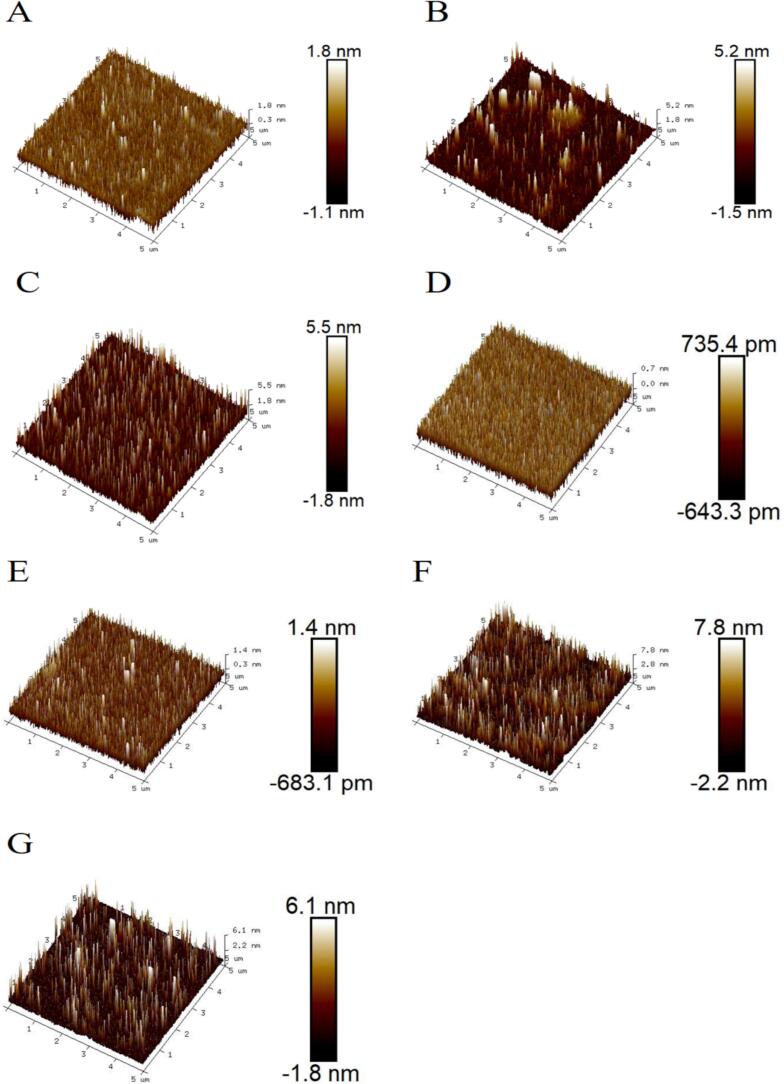
The nanostructure of WPI-LBP complexes with different heat treatment times.

Fig. 6 illustrates how varying heat treatment durations affect the nanostructure of WPI-LBP complexes. The WPI-LBP mixture was rougher (Ra = 0.592 nm) and more aggregated than WPI (Fig. 6A and B). In contrast to the initial roughening induced by LBP, moderate heating (30 min) generated a uniform, densely packed nanostructure with minimal roughness (Ra = 0.165 nm), while shorter heating (15 min) represented an intermediate state with increased roughness (Ra = 0.716 nm) but greater size uniformity. (Fig. 6C and D). This outcome might be due to moderate heat treatment, where introduced polysaccharide chains enhance steric hindrance, preventing excessive aggregation and forming tightly packed, uniformly sized aggregates (Shu et al., 2018). This result differs from previous studies' Ra values, possibly due to using preheated ultrapure water for sample dilution during AFM analysis, leading to more uniform samples Wang et al., 2025). Further extension of heating time (60–180 min) reversed this trend, leading to a progressive coarsening of the nanostructure. This was characterized by the emergence and growth of large aggregates, a concomitant rise in surface roughness (Ra from 0.205 to 0.428 nm), and eventual agglomerate formation, consistent with the SEM findings. This trend is attributed to a transition from the initial, beneficial cross-linking that inhibits aggregation, to the eventual damage of this structure under prolonged heat, which reduces steric hindrance and promotes aggregation (Shu et al., 2018). Additionally, studies show that protein aggregate formation is related to hydrogen bonding and electrostatic interactions within the system (Huang et al., 2017; Wang, Rao, et al., 2024). This further supports the results of solubility, CD, and XRD, indicating that different heating times also affect intramolecular hydrogen bonding in WPI-LBP complexes.

#### 3.6.6 DSC analysis

The DSC results of WPI and WPI-LBP complexes with different heat treatment times are shown in Fig. 3B. Compared with native WPI, the addition of LBP alone reduced its thermal denaturation temperature (T_d_) from 87.54 °C to 75.20 °C, and when heat-treated for 15 min, the T_d_ further decreased to 67.98 °C. This indicates that the initial maillard reaction at room temperature (DG = 10.41 %) to the intensified grafting induced by heating (DG = 23.49 %) altered the native structure of WPI, thereby reducing its T_d_. Notably, when the heat treatment time was extended to 30 min, the T_d_ of the WPI-LBP complex increased to 77.42 °C. The rise in T_d_ is attributed to the formation of highly cross-linked covalent aggregates induced by the maillard reaction, a conclusion supported by SDS-PAGE (enhanced high-molecular-weight bands) and changes in free sulfhydryl content. When the heat treatment was further prolonged to 60, 120, and 180 min, the T_d_ decreased again and continued to fluctuate at relatively low levels. This suggests that excessive heat treatment leads to the degradation of the complexes, resulting in the formation of structurally disordered heterogeneous aggregates, which is consistent with the subsequent decrease in DG value and the structural damage observed in SEM images.

### 3.7 The optimal processing window (15–60 min) yields a cohesive and functional complex

**Fig. 7:**
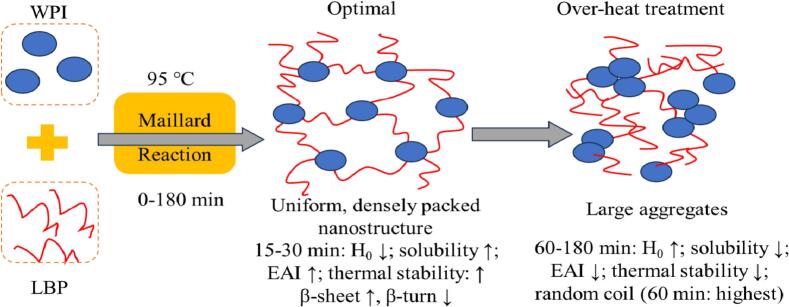
Schematic illustrating the mechanism of heat-induced structure-function changes in WPI-LBP complexes.

Our collective findings demonstrate that a thermal processing duration of 15 to 60 min constitutes an optimal window for constructing WPI-LBP complexes with superior functional properties. This conclusion is robustly supported by multidimensional evidence spanning molecular structure, microscopic morphology, and macroscopic functionality (Fig. 7). Within this window, the complexes developed a uniform and densely packed nanostructure, which established the physical foundation for enhanced functionality. AFM revealed minimal surface roughness (Ra = 0.165 nm at 30 min, Fig. 6D-D‘), while SEM visually illustrated the structural evolution: after short-to-moderate heat treatment (15–30 min), the complexes progressively developed smoother surfaces and denser, cross-linked network structures (Fig. 5C-C‘and D-D'). This structural optimization stemmed from moderate heat-induced interactions between WPI and LBP, specifically evidenced by a significant reduction in H₀, with the lowest value reaching 1897.9 at 30 min (Xing et al., 2024) and specific secondary structure rearrangements (CD spectroscopy indicated the highest β-sheet content of 46.80 % and the lowest β-turn content of 5.90 % at 30 min) (Wang et al., 2025). These changes suggest a controlled unfolding and restructuring of the protein molecules, leading to the burial of hydrophobic groups via intermolecular interactions (Li et al., 2025) and the formation of a new, more stable hydrogen-bonding network (Jiang et al., 2021; Li, Lai, et al., 2024). Consequently, this optimized structure directly translated into exceptional solubility and EAI, both of which reached their optimal values at 30 min. The evidence suggests that this peak performance was mediated by the grafting of LBP onto partially unfolded WPI, a process that enhanced hydrophilicity and provided steric hindrance through covalent or non-covalent bonds (Shu et al., 2018). This mechanism effectively inhibited excessive protein aggregation and promoted the formation of small, uniform aggregates. However, extending the heating time beyond 60 min disrupted this delicate balance. Excessive thermal stress caused severe protein unfolding, leading to re-exposure of hydrophobic groups (increased H₀) (Qu et al., 2018) and a rise in random coil content (reaching its maximum of 39.37 % at 60 min), and the disruption of the previously uniform nanostructure (increased Ra and formation of large aggregates) (Shu et al., 2018). Furthermore, SEM results revealed a progressive microstructural breakdown, which culminated in severe disruption with extended heating (60–180 min). These adverse changes ultimately resulted in the decline of solubility and EAI (Qu et al., 2018; Wang et al., 2025). In summary, thermal treatment for 15–60 min successfully facilitates the precise modulation of protein-polysaccharide interactions, leading to the construction of a structurally compact, interfacially stable, and functionally enhanced complex at the molecular, microscopic, and macroscopic levels.

## 4 Conclusion

This study analyzed the effects of different heat treatment times on the functional properties and structure of WPI-LBP complexes, as well as the impact on their interaction mechanisms by regulating the heat treatment time. The results showed that moderate heat treatment times (15 to 60 min) effectively promote cross-linking between WPI and LBP, as confirmed by SDS-PAGE. Solubility, emulsifying properties, and thermal stability of WPI-LBP complexes peaked at 30–60 min of heat treatment, with H_0_ reaching its lowest at 30 min. Free SH content decreased and then increased with heat treatment time, hitting a minimum at 15 min. The analysis of water distribution suggests that different heat treatment times may induce changes in water distribution by regulating the structure. CD results indicated that different heat treatment times affect the secondary structure of WPI-LBP complexes, with the highest β-sheet (46.80 %) and lowest β-turn (5.90 %) at 30 min, and the lowest α-helix (8.97 %) and highest random coil (39.37 %) at 60 min. XRD results showed varying impacts on the crystal structure of WPI-LBP with different heat treatment times. Additionally, SEM and AFM results revealed that moderate heat treatment times favor the formation of microstructures and nanostructures that enhance functional properties, while prolonged heat treatment increases surface roughness, aggregate size, and can even damage some network structures. The structural changes were further supported by 10.13039/100009131DSC analysis. Furthermore, the above results also indicate that the WPI-LBP complexes are predominantly stabilized by covalent, hydrogen, hydrophobic, and electrostatic interactions, with the heat treatment time playing a critical role in modulating these interactions. These findings suggest that optimizing heat treatment time is crucial for maintaining the structural and functional properties of WPI-LBP complexes, providing a theoretical basis for potential applications in food processing, such as in the development of ingredients for emulsified systems or as functional protein supplements.

## CRediT authorship contribution statement

**Shaomin Zheng:** Visualization, Methodology, Investigation, Formal analysis, Data curation. **Fangyan Huang:** Visualization, Methodology, Investigation, Formal analysis, Data curation. **Bingqiang Wang:** Visualization, Methodology, Investigation, Formal analysis, Data curation. **Jinjing Yang:** Methodology, Investigation. **Huan Han:** Methodology. **Leiwen Xiang:** Writing – review & editing, Validation, Supervision, Methodology, Conceptualization. **Hailin Wang:** Writing – review & editing, Writing – original draft, Visualization, Supervision, Project administration, Methodology, Investigation, Funding acquisition, Formal analysis, Data curation, Conceptualization.

## Declaration of competing interest

The authors declare that they have no known competing financial interests or personal relationships that could have appeared to influence the work reported in this paper.

## Data Availability

Data will be made available on request.

## Appendix A Supplementary data

Supplementary material Image 1
