# Supplementary material for: Deciphering interaction mechanisms in heat treatment time-modulated whey protein isolate-Lycium barbarum polysaccharide complexes: perspectives from structural-functional evolution
Source: Food Chem X. 2025 Dec 22;33:103440. doi: 10.1016/j.fochx.2025.103440 (PMC12807803; doi:10.1016/j.fochx.2025.103440)
Supplement: Supplementary file 1 — Supplementary material [file mmc1.docx]

**Supplementary material**

**Table S1** The relaxation time and associated peak areas of WPI-LBP complexes with various heat treatment time.

| Samples | T_21_ (ms) | T_23_ (ms) | S_21_ (%) | S_23_ (%) |
| --- | --- | --- | --- | --- |
| WPI | 0.766 | 1979.167 | 1.917 | 98.083 |
| 0 min | 1.06 | 1320.088 | 1.527 | 98.473 |
| 15 min | 0.511 | 880.488 | 1.255 | 98.745 |
| 30 min | 1.351 | 1035.322 | 0.774 | 99.226 |
| 60 min | 0.601 | 1035.322 | 1.387 | 98.613 |
| 120 min | 0.471 | 1035.322 | 0.251 | 99.749 |
| 180 min | 0.977 | 690.551 | 0.736 | 99.264 |
